# Analysis of Electric Motor Magnetic Core Loss under Axial Mechanical Stress

**DOI:** 10.3390/s20236818

**Published:** 2020-11-29

**Authors:** L. Ashok Kumar, Bagianathan Madhan Raj, Varadarajan Vijayakumar, Vairavasundaram Indragandhi, Vairavasundaram Subramaniyaswamy, Hamid. R. Karimi, Kalyana C. Veluvolu

**Affiliations:** 1 PSG College of Technology, Coimbatore 641004, India; lak@eee.psgtech.ac.in; 2 Specialist Motors, ELGI Equipment Ltd., Coimbatore 641005, India; madhanrajb@magna.com; 3 Department School of Computer Science and Engineering, The University of New South Wales, Sydney, NSW 2052, Australia; v.varadarajan@unsw.edu.au; 4 Vellore Institute of Technology, Vellore 632014, India; indragandhi.v@vit.ac.in; 5 School of Computing, SASTRA Deemed University, Thanjavur 613401, India; swamy@cse.sastra.edu; 6 Department of Mechanical Engineering, Politecnico di Milano, 20156 Milan, Italy; hamidreza.karimi@polimi.it; 7 School of Electronics Engineering, Kyungpook National University, Daegu 41566, Korea

**Keywords:** ansys maxwell software, axial pressure, iron loss, magnetic core, mechanical stress

## Abstract

The electrical machine core is subjected to mechanical stresses during manufacturing processes. These stresses include radial, circumferential and axial components that may have significant influence on the magnetic properties and it further leads to increase in iron loss and permeability in the stator core. In this research work, analysis of magnetic core iron loss under axial mechanical stress is investigated. The magnetic core is designed with Magnetic Flux Density (MF) ranging from 1.0 T to 1.5 T with estimated dimensions under various input voltages from 5 V to 85 V. Iron losses are predicted by the axial pressure created manually wherever required and is further applied to the designed magnetic core in the range of 5 MPa to 50 MPa. Finite element analysis is employed to estimate the magnetic core parameters and the magnetic core dimensions. A ring core is designed with the selected dimensions for the experimental evaluation. The analysis of iron loss at 50 Hz frequency for non-oriented electrical steel of M400-50A is tested experimentally using the Epstein frame test and force-fit setup test. Experimental evaluation concludes that the magnetic core saturates when it reaches its knee point of the B-H curve of the chosen material and also reveals that the axial pressure has a high impact on the magnetic properties of the material.

## 1. Introduction

Iron loss is often considered as the main source of error in the prediction of the motor efficiency. During the manufacturing of an electrical motor, some mechanical stress are often produced due to stamping, punching and stacking in axial or radial directions. It leads to deterioration of the magnetic properties in the core materials and leads to iron loss in the core thereby decreasing the efficiency of the electrical machine [1,2,3,4]. The main causes of stress are the stamping of the laminations, the clamping of the laminated core, and the shrink or press fitting of the core into a frame. Various investigators have reported on the effects of stress on the iron loss. However, even for classical ac sinusoidal flux machines, the difference between iron loss predictions based on specific iron loss density values supplied by lamination steel manufacturers and the measured iron loss cannot be attributed solely to the effect of stress [5,6]. For example, the complex core geometry and MMF distribution of an electrical machine can cause significant harmonics in local flux density waveforms, which vary with the operating condition, and the resulting harmonic iron loss can account for a substantial portion of the total iron loss [7,8,9]. However, this can now be quantified at the design stage. The past decades have witnessed numerous developments [10] in optimization and improvement of rotating electrical machine design and this has led to the development of models that evaluate the losses in electrical machines. The mechanical joining and the fusion welding of the electrical steel laminations and its effects on the magnetic properties of the designed core are well reviewed in [5]. The review findings show that joining and welding increase the eddy current loss in the designed core [5]. The measure of magnetic properties of welded electrical steel laminations are studied in [11]. An equivalent circuit based mathematical model was proposed in [12] for the estimation of eddy current losses in the welded electrical steel laminations.

In [13], a new optimization method is proposed to enhance the performance of the switched reluctance motor drive systems under multiple operating conditions. A new multi-level optimization strategy for the multi-objective optimization of an interior permanent synchronous motor (IPMSM) by considering the Pearson correlation coefficient analysis and cross-factor variance analysis is developed in [14]. Core losses of a novel 16/10 segmented rotor switched reluctance motor (SSRM) were calculated by a nonlinear lumped model in [15]. The proposed model uses the method of energy conversation for calculation of hysteresis, eddy current losses and anomalous losses.

An experimental method to characterize the magnetic properties of Grain Oriented Electrical Steel in the rolling direction was proposed in [16]. In [17], a stator core shape design method is proposed to improve power density of a surface-mounted permanent magnet (SPM) motor. The proposed design method improves the power density of a motor by reducing its weight without decreasing the torque and keeping the winding regions constant. Recent research results in the current state of the art [18,19] justify that the iron loss is stress-dependent and the mechanical stresses have an adverse effect on the magnetic properties of the electrical iron lamination sheet. The main causes of stress are stamping of the laminations, clamping of the laminated core, and shrinking or press-fitting of the core into a frame. These stresses lead to increase iron loss and permeability in the stator core. Therefore, the dependence of the core losses on multi-axial stresses should be studied extensively in order to design effective machines and to also evaluate current ones with greater precision.

The work in [20] described analytical and numerical techniques that have been developed for predicting the effect of compressive stress on the iron loss density in the laminating material. The work further demonstrated and analyzed the calculation of iron losses by finite element method for a permanent magnet brushless DC motor. In [21], a new method is developed to calculate the iron losses of permanent magnetic machines by considering hysteresis loops affected by multi-axial stress. The hysteresis model in the governing equation of finite-element analysis (FEA) is employed to estimate the effects of multi-axial stress on electrical steel sheets. The experimental and numerical characteristics of the magnetic behaviour of thin steel sheets under mechanical stress was dealt with in [22]. The magnetic properties of the testing material under pulsating magnetic flux and compressive stress are analyzed in this paper.

In [23], the effect of magneto-mechanical and magneto-crystalline anisotropies in a test application was investigated by coupling a multi scale magneto-mechanical model with a finite element approach. The effect of multi-axial stress on the eddy current and hysteresis losses in electrical sheets are analyzed in [24]. Experimental results validate that an equivalent stress approach is effective for core loss estimation in rotating machines. A finite element approach is presented in [25] which accounts for the core loss in a permanent magnet motor fed inverter that is generated due to the high order harmonics caused by Pulse width modulation (PWM). This framework was used to predict the iron losses of the electrical machines considering the skin effect and the presence of the minor hysteresis loops under high order harmonics. The iron loss was analyzed by stress and EMF analysis. The model for the evaluation of stress in the transverse direction with respect to flux density of isotropic materials with magneto-mechanical problems was proposed in [26]. This proposed model was employed to identify the permeability variations due to stress. In [27], a coupled magnetomechanical model is proposed to explore the correlation between the asymmetrical variation of the magnetostriction and the B–H characteristic with applied coaxial stress. Results show that the area between the B–H curve was under stress and the stress increases with the permeability.

Further, an extension to the Jiles–Atherton hystersis model, a multiscale modelling of the anhysteretic magnetization, is proposed in [28] by considering the mechanical stress and the crystallographic texture effects. To numerically analyze the behaviour of iron sheets under biaxial stress, a novel single sheet tester is proposed in [29]. The approach implemented the finite element (FEM) model in the tester device by considering the coupled magneto-mechanical model that can be identified using stress dependent anhysteretic B–H curves. It has been demonstrated that the uniform field distribution and desired in-plane stress tensor in the center point of any sample iron sheet can be obtained by applying the suitable mechanical loading with the proposed tester device.

To model the permeability changes in ferromagnetic materials due to mechanical loading, an equivalent stress–strain approach is proposed in [30]. Furthermore, the proposed model is used for transforming the complex multiaxial mechanical loading into equivalent uniaxial loading parallel to the magnetic field and, consequently, the permeability of the material can be predicted using the uniaxial measurements. However the proposed framework failed to estimate the stress when the materials are exposed to the bi-compressive stress. Permanent magnet synchronous generator (PMSG) system performance metrics are explored in [31]. In contrast to existing works, finite element analysis (FEA) was employed to analyze the machine as whole and that resulted in a detailed electromagnetic study with accurate results. The complex function of permeability was used for the first time to model the impact of stress in [32]. The developed scheme in [32] is consistent with numerical analysis and significantly decreases the computational time. However, the suggested model is only applicable to steady state or cyclic fields and identifies only the reversible impacts. A new technique was developed to estimate the Iron losses from measurements on fully assembled stators in [33]. The proposed technique does not require any additional samples as it utilizes fully assembled stators. Results from Epstein tests show that estimated iron losses are twice as large at 10 kHz. This stresses the need to integrate industrial impact on iron losses at higher frequency. A simple hysteresis model coupled with tie-stepping finite element analysis is developed in [21] to analyze the iron loss of permanent-magnet machines. The validity of the technique is experimentally verified by key material studies under the configuration of multi-axial loading. The influence of sample deformation was calculated on the magnetization curve and overall core energy losses, both during mechanical loading and release. It is understood that, with higher sample bending under mechanical load, the magnetic properties significantly degrade. Furthermore, in [34], it was observed that the magnetic properties are further when the mechanical load is removed.

As iron loss is the major source of error in the prediction of motor efficiency, this work aims to analyze iron loss under different pivotal mechanical stresses. The test setup is intended to dissect the impact of stacking pressure due to force fit in a ring core loop and to also create 2D finite element examination for the prediction of iron loss. In this work, the force fit set up is developed successfully for a ring core loop to analyze the iron loss with and without the mechanical stress. This paper analyzes the magnetic core iron loss under axial mechanical stress. The magnetic core is designed with magnetic flux density (MF) in the range of 1.0 to 1.5 T. Iron losses are predicted by the axial pressure created manually wherever required and is further applied to the designed magnetic core in the range of 5 to 50 MPa. Analysis is conducted through simulations with Ansys Maxwell Software and magnetic core dimensions are selected which meet the required specifications. Based on the finalized dimensions, the ring core is manufactured for the experimental evaluation. The applied pressure is measured by using a strain gauge. The analysis of iron loss at 50Hz frequency for non-oriented electrical steel of M400-50A is tested experimentally using the Epstein frame test and force-fit setup test. The rest of this paper is organized as follows: Section 2 presents the design methodology for magnetic rectangular and ring cores. Simulation results are presented in Section 3, and Section 4 presents the experimental results. Section 5 concludes the paper.

## 2. Methodology

**Figure 1:**
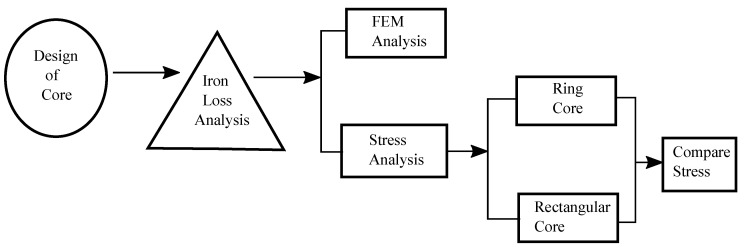
Block diagram of the proposed system.

**Figure 2:**
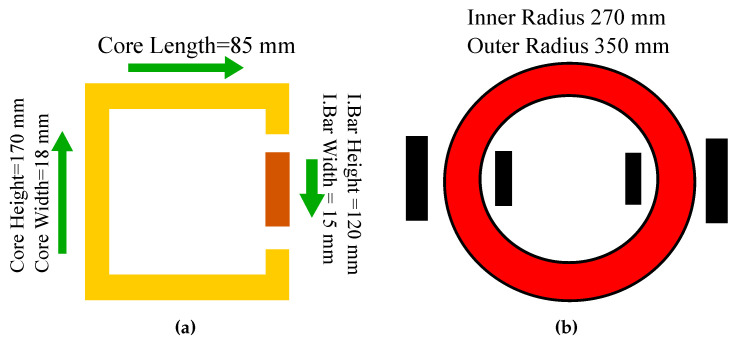
(**a**) Rectangular core loop, (**b**) Ring core loop.

This section presents the design of Magnetic cores for the analysis of core loss as shown in Figure 1. The cores are first designed in Ansys Maxwell software and evaluated through simulations in the first stage. The magnetic core is designed with a dimension of 170 mm × 85 mm by using a C-shaped and I-shaped core with an air gap of 0.5 mm. Figure 2a,b present the designs of the magnetic rectangular core and ring core loop, respectively.

Table 1 and Table 2 present the input specifications of the magnetic rectangular core and ring core loop for design calculations. The dimensions predicted for the magnetic core for the ring and rectangular loop will have the capability to allow MF in the range of 1.0 T to 1.5 T under the various input voltages. The magnetic core is designed with the software Ansys Maxwell. The core loss computing algorithm can be summarized as follows: Create the core as it depends on the shapes like circle and rectangles by using the polyline from the selected items. Create the excitation for the coil by creating a circuit editor. Hence, set the model depth which it is calculated by using the analytical calculation in modeler units. Then set the eddy effect and core loss effect for analysis. After that set the boundary condition to zero in vector potential. The final step is to set the solution setup and check the analysis for each of them.

## 3. Simulation Results

In this section, simulation results for the ring core are first discussed followed by the results for the rectangular core. The observations from the 2D analysis and field overlays for ring core are provided in Table 3. It lists the parameters like the number of turns, primary resistance, primary inductance, secondary resistance and secondary inductance. These values are further used in the circuit editor to excite the coil.

**Figure 3:**
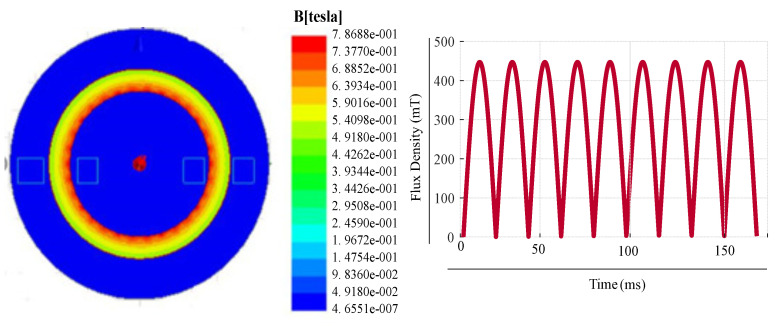
Ring core loop for input of 20 V.

**Figure 4:**
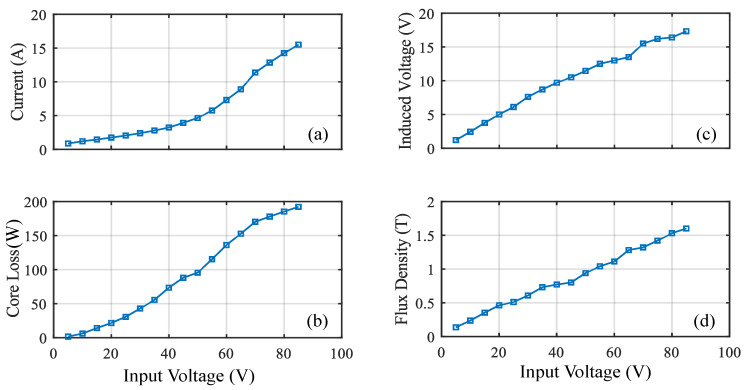
Performance of ring core: (**a**) current (**b**) core loss (**c**) induced voltage (**d**) flux density.

Figure 3 shows the ring core with flux density of 0.45 T together with the respective flux density plot for input voltage of 20 V. The usage of pointer in the ring loops confirms the MF in the core. From the results, it can be observed that the MF starts saturating when it reaches its knee point of the B–H curve of the chosen material. The simulation results for a ring core under different input voltages without any mechanical stress are shown in Figure 4. This resembles to an actual core loss analysis in FEM. Table 4 shows the analysis of the results for an input voltage of 60 V. From the analysis, the core loss obtained is 136.1 W with the MF of 1.1 T and current of 7.31 A.

### 3.1. Result for Field Overlays of Rectangular Core

**Figure 5:**
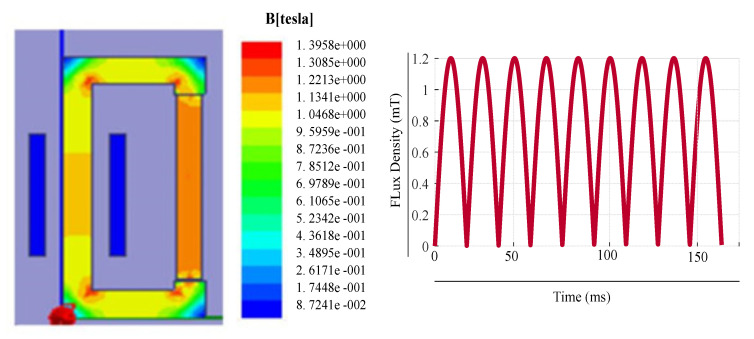
Rectangular core for input of 50 V.

**Figure 6:**
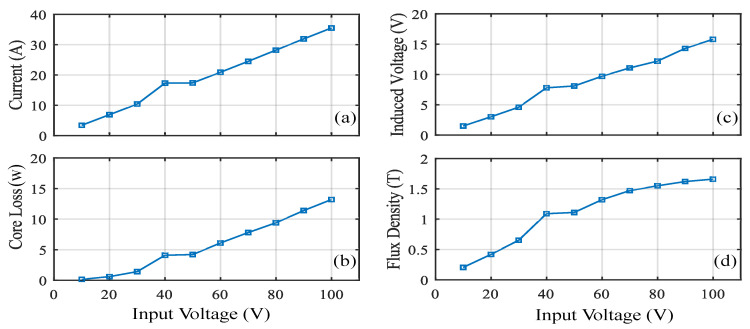
Performance of rectangular core: (**a**) current (**b**) core loss (**c**) induced voltage (**d**) flux density.

Figure 5 Shows the rectangular core with MF of 1.1 T for an input voltage of 50 V. Table 5 shows the analysis of the results for an input voltage of 90 V. The core loss obtained is 14.3 W with the MF of 1.6 T and current of 31.9 A. Figure 6d shows the relation between the input voltage and MF. From the figure, it can be observed that the MF increases linearly and it depends on the input voltage. But the core saturates when it reaches the knee point for the chosen material M400-50 A. Figure 6a shows the relation between the input voltage and current. The figure shows that the current will have a maximum of 35.5 A for the input voltage of 100 V. Hence, the current of this magnitude satisfies our requirement. Figure 6b shows the relation between the Input voltage versus core loss. The core loss obtained is 13.2 W without any axial mechanical stress for the maximum input voltage of 100 V. The induced voltage obtained is 15.8 V for the input voltage of 100 V.

### 3.2. Comparison for Various Number of Turns

**Figure 7:**
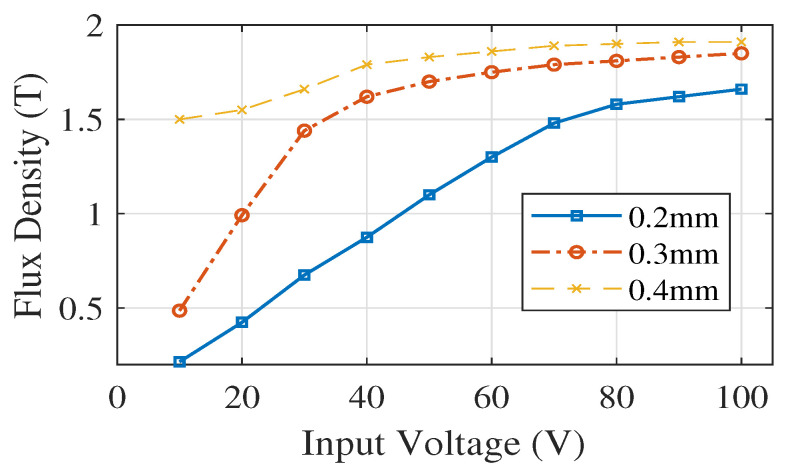
Comparison analysis of current for various number of turns.

**Figure 8:**
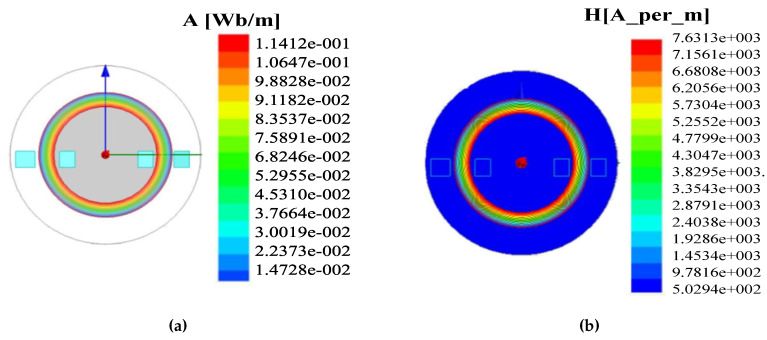
(**a**) Ring core Flux lines (Wb) (**b**) magnetic field intensity (H/m).

Simulation results for comparison of current for various combinations of primary and secondary turns are presented in Figure 7. The primary and the secondary turns considered for analysis are (24, 8); (32, 16); (81, 18). It can be observed that the primary and the secondary turns (24, 8) generates the required MF of 1 T to 1.5 T with a current range from 0.8 A to 16 A. Figure 8a shows the flux lines of the ring core. It illustrates that flux flows into the ring core with expected MF of 1 T to 1.5 T under the different input voltages. Magnetic flux intensity analysis for the ring core loop is shown in Figure 8b and it can be seen that it reaches up to 0.7 (A/m).

**Figure 9:**
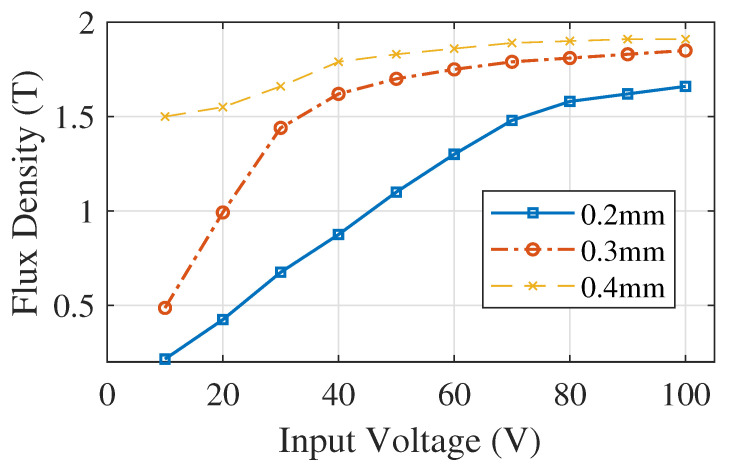
Current for various diameter selections for core under 25 turns.

**Figure 10:**
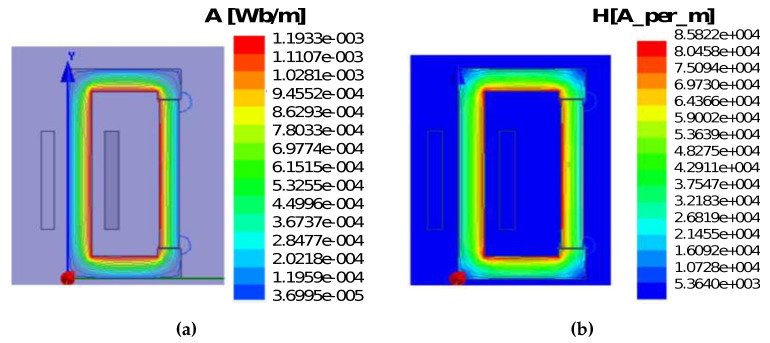
(**a**) Rectangular core Flux lines (Wb); (**b**) magnetic field intensity (H/m).

Figure 9 presents the current analysis for various diameter selections (0.2 mm, 0.3 mm, 0.4 mm) for rectangular core under 25 turns. From the analysis, we can see that 25 number of turns do not meet our requirement. The analysis shows that the rectangular magnetic core starts to saturate when it reaches a certain input voltage. But the current value varies and depends upon the wire diameter and number of turns. Figure 10a presents the flux lines of the rectangular core. It illustrates that flux flows into the rectangular core with an expected MF in the range of 1 T to 1.5 T for different input voltage selections. Figure 10b depicts the Magnetic flux intensity analysis for the rectangular core and its magnitude reaches up to 8.5 (A/m).

## 4. Experimental Results

**Figure 11:**
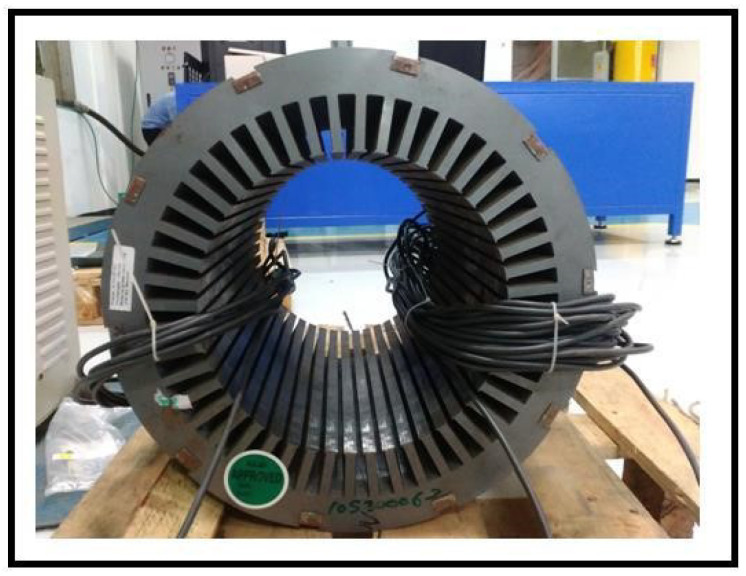
Designed ring core.

Although detailed analysis and simulation results are presented for both rectangular and ring cores, the experimental evaluation is only limited to ring core in this section. Based on the predicted dimensions from the simulation analysis, a ring core is designed as shown in Figure 11. The MF capability is evaluated from 1 T to 1.5 T. Figure 11 presents the designed ring core loop with primary and secondary turns of 24 and 8 turns respectively. The parameter values for the designed core are tabulated in Table 3.

The input voltage ranges from 5 V to 75 V at 50 Hz for the non-oriented electrical steel of M400-50A. The pressure applied ranges from 5 Pa to 50 Pa for various input voltage selections. The pressure can be measured by using a strain gauge. The MF can be measured by a search coil as required and it ranges from 1 T to 1.5 T. The power quality analyzer is used for the measure of output current and voltage. Experiments are performed under loose and tight lamination by applying the pressure.

### 4.1. Ring Core Loop Test Under Loose Lamination (Without Mechanical Stress)

**Figure 12:**
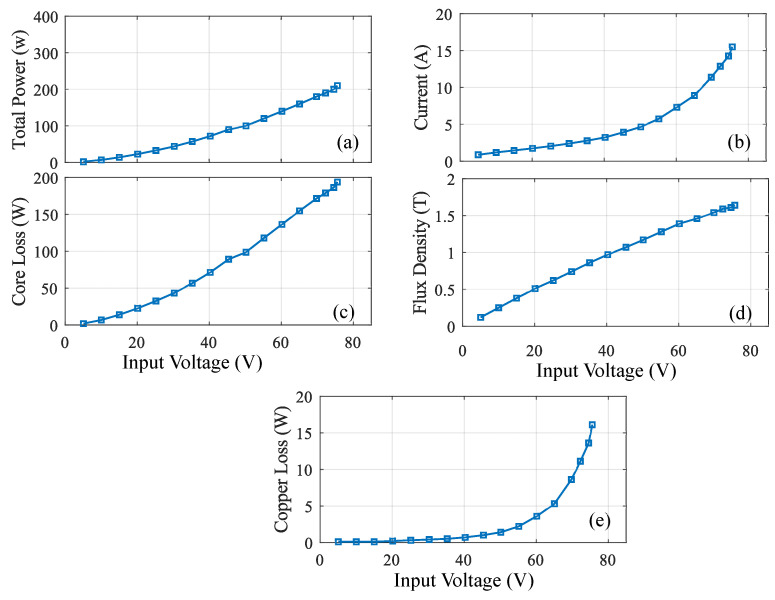
Ring core loop test under loose lamination (without stress): (**a**) total power (**b**) current (**c**) core loss (**d**) flux density (**e**) copper loss.

Experimental results of ring core loop under loose lamination are shown in Figure 12. From the results it can be observed that the primary voltage from 5 V to 75 V is applied under the different pressure level from 5 Pa to 75 Pa. The change in MF can be observed to be in the range of 1 T to 1.6 T. Hence the MF starts saturating when it reaches the MF of 1.6 T. Due to the change in MF, core loss is produced with application of hydraulic pressure under loose lamination. Figure 12b shows the relation between the input voltage versus current. The figure shows that the increase in the magnitude of the current depends on the input voltage. The current values of the ring core loop reaches up to 16 A for the maximum input voltage of 75 V under the mechanical pressure. Figure 12d shows the relation between the input voltage versus MF. The MF ranges from 1 T to 1.6 T for different input voltages. Figure 12c shows the relation between the input voltages versus core loss. It can be observed that the increase in the core loss depends on the input voltage. The core loss in the ring core loop reaches up to 193.6 W for the maximum input voltage of 75 V under the mechanical pressure. Figure 12a shows the relation between the input voltage versus total power on the secondary side. The increase in the total power depends on the input voltage.

### 4.2. Core Ring Loop Test Under Tight Lamination (Under Mechanical Stress)

**Figure 13:**
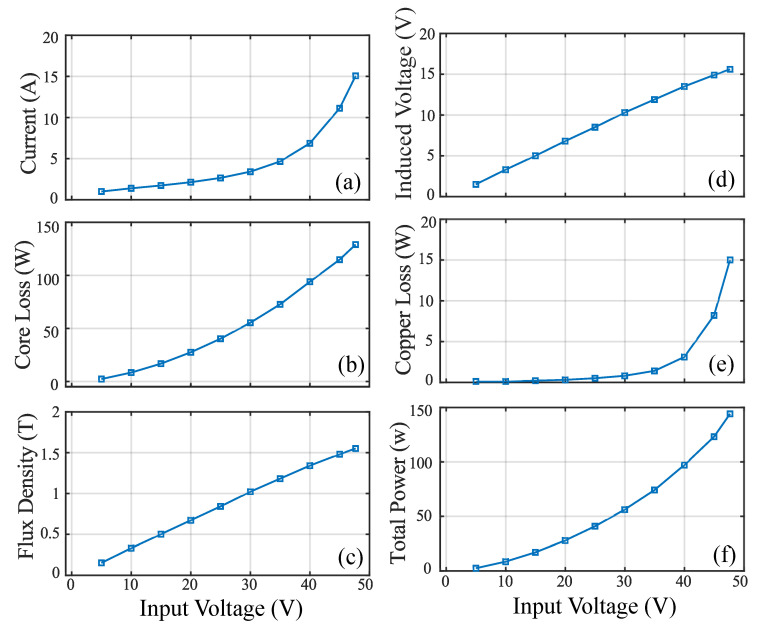
Ring core loop test under tight lamination (with stress): (**a**) input voltage vs. current (**b**) input voltage vs. core loss (**c**) input voltage vs. flux density (**d**) input voltage vs. induced voltage (**e**) input voltage vs. copper loss (**f**) input voltage vs. total power.

For the ring loop core with tight lamination, the experimental results obtained are shown in Figure 13. The hydraulic pressure in the range of 5 Pa to 75 Pa is applied to the core. Figure 13a shows that the input voltage increases with the increase in the current. The MF varies in the range of 1 T to 1.6 T for various selections of input voltage as shown in Figure 13c. The core loss reaches up to 193.6 W for the maximum input voltage of 47 V under the mechanical pressure. Figure 13d shows the relation between the input voltage versus induced voltage.

**Figure 14:**
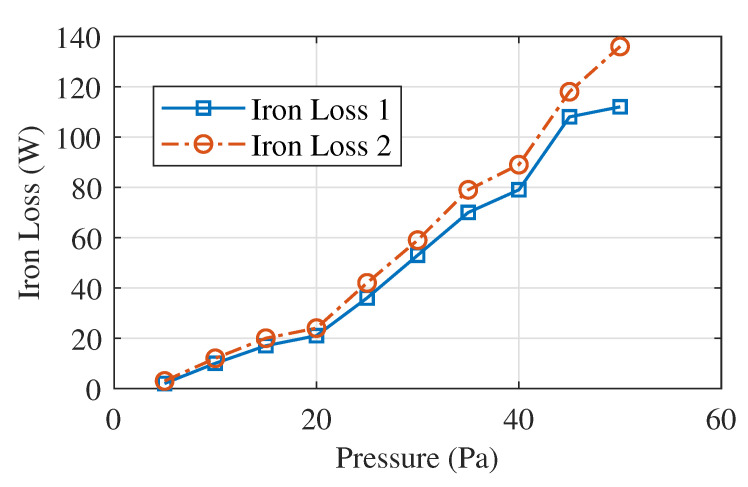
Iron core loss with and without stress.

For comparison, the core loss obtained for the case of presence and absence of the mechanical stress are shown in Figure 14. It clearly shows that the core loss is increased when the mechanical pressure is applied. Under the loose lamination, the ring core loss at 1.5 T core loss density is 4.5 W/kg. Whereas for the tight lamination, the ring core loss at 1.5 T core loss density is 3.5 W/kg.

### 4.3. Epstein Frame Test

**Figure 15:**
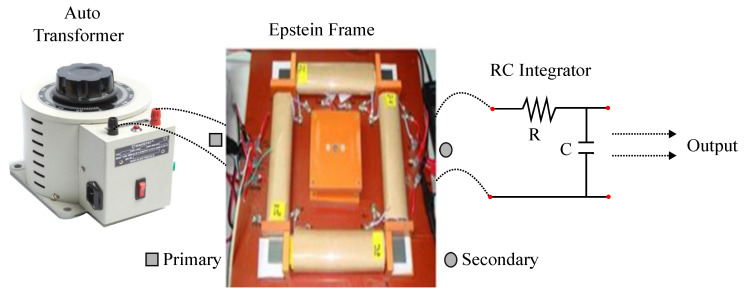
Epstein frame test for evaluation of core loss.

The general Epstein frame test shown in Figure 15 is employed to evaluate the core loss in a lamination sheet by using a global core loss predictor with the help of global tester software. Epstein frame test is a simple way to find the core loss of the material. The primary winding of the Epstein frame is connected to a 1-ϕ auto transformer with an input of 230V, 50Hz as shown in the circuit diagram. The voltage is analogous to the magnetic field around the resistance associated with the main. The secondary side of the Epstein frame is connected to a RC integrator. The CRO probe is connected across the capacitor to obtain B–H curve. With an increase in voltage, we can observe a parabola on the display of the CRO which slowly converts into a leaf like structure (B–H Loop) with the increase in voltage. The unit enables one to trace the B-H loop (hysteresis) of a ferromagnetic specimen using a Cathode Ray Oscilloscope (CRO). A measurement of the area of the loop lead to the evaluation of energy loss in the specimen. The flux density is measured by using the flux meter and WT3000 power meter is used to determine the losses in the test sample during the measurement.

The primary voltage in the range of 5 V to 50 V is applied and the change in magnetic flux density can be observed to be in the range of 1 T to 1.6 T with the estimated dimensions. As iron losses can be predicted by the axial pressure where it is created manually which and applied to the designed magnetic core ranging from 5 MPa to 50 MPa for the analysis of iron loss at 50 Hz frequency. The measured current reaches to 15 A and total maximum energy loss reaches 129 W. The eddy current loss is estimated using the obtained maximum flux density. Neglecting the end portions of the path, the magnitude of eddy current is estimated. From the area enclosed by B–H the hysteresis losses are estimated manually.

## 5. Conclusions

The stress investigation on a ring core is performed using the force fit setup and the iron loss under different axial stress is examined. The Epstein frame test is proposed for a rectangular-shaped core. From the outcomes of force fit test, it reveals that the iron loss is stress-dependent and hence mechanical stress will deteriorate the B–H of the material M400-50A. The magnetic properties under the axial stress until 50 Pa or more are measured by using the laminated specimen of non-oriented electrical steel M400-50 A. The obtained result shows that there is a decrease in the relative permeability and an increase in the iron loss with the compressive stress. Also, high deterioration of magnetic properties is observed at the stress value of less than about 50 Pa in the case of the non-oriented electrical steel M400-50A. Both hysteresis loss and eddy current loss increase with the increase of stress. The increase of hysteresis loss is due to the increase of coercivity under the stress.

MDPI stays neutral with regard to jurisdictional claims in published maps and institutional affiliations.

## Figures and Tables

**Table 1:** Parameters for rectangular core.

Parameters	Values
Height of core	170 mm
Length of core	85 mm
Core width (Axial length)	18 mm
Width 1 of core	24.5 mm
Width 2 of core	6.5 mm
Inner length of core	134 mm
Inner height of core	49 mm
Bar height	120 mm
Bar width	15 mm
Diameter of wire	0.2 mm
Stacking length of core	30 mm
Density of material	7700 kg/m^3^
Stacking factor	0.9
Input voltage	100 V
Frequency	50 Hz
Number of turns	50 Turns

**Table 2:** Parameters for ring core loop.

Parameters	Values
Outer radius	350 mm
Inner radius	270 mm
Inner diameter	175 mm
Outer diameter	135 mm
Height	350 mm
Number of primary turns	24 Turns
Number of secondary turns	8 Turns
Input voltage	50 V
Stacking length of the core	30 mm
Stacking factor	0.9
Frequency	50 Hz
Diameter of wire	1.3 mm
Density of material	7700 kg/m^3^
Resistivity of copper	$1.75\times 10^{-5}$
Permeability of free space	$4\times 3.14\times 10^{-7}$
Magnetic field intensity	200 A/m

**Table 3:** Parameters for ring core.

Parameters	Primary	Secondary
No.of turns	24	8
Resistance	0.06 Ω	0.025 Ω
Inductance	3.8 mH	0.397 mH

**Table 4:** Analysis for input voltage of 60 V.

Parameters	Values
Primary voltage	60 V
Current	7.31 A
Secondary induced voltage	13 V
Core loss	136.1 W
MF	1.1 T

**Table 5:** Result analysis for input voltage of 90 V.

Parameters	Values
Primary input voltage	90 V
Current	31.9 A
Induced voltage	11.4 V
Core loss	14.3 W
MF	1.6 T
